# Method Validation and Establishment of Reference Intervals for an Insulin-like Growth Factor-1 Chemiluminescent Immunoassay in Cats †

**DOI:** 10.3390/vetsci10090575

**Published:** 2023-09-15

**Authors:** Arne Güssow, Sabine Thalmeier, Ruth Gostelow, Judith Langenstein, Gesine Foerster, Natali Bauer, Katarina Hazuchova

**Affiliations:** 1 Clinic for Small Animals (Internal Medicine, Clinical Pathology and Clinical Pathophysiology), Justus-Liebig-University, 35392 Giessen, Germany; 2 Department of Clinical Science and Services, The Royal Veterinary College, Hatfield AL9 7TA, UK; 3 SYNLAB.vet GmbH, 86156 Augsburg, Germany

**Keywords:** IGF-1, feline, radioimmunoassay, precision, diabetes mellitus, hypersomatotropism, reference range, cut-off

## Abstract

**Simple Summary:**

Hypersomatotropism results from excess growth hormone production by a pituitary tumour and represents an important underlying cause of diabetes mellitus in cats. Diagnosis of hypersomatotropism is mainly based on the measurement of insulin-like growth factor-1 (IGF-1), ideally in combination with imaging of the head. The most well-validated assay (radioimmunoassay), however, requires facilities approved to handle radioactivity and is costly. This study validated an alternative method for IGF-1 measurement in cats, a chemiluminescence assay, and compared it to the radioimmunoassay. It also established a reference interval for IGF-1. This will increase availability of IGF-1 measurement and facilitate diagnosis of hypersomatotropism.

**Abstract:**

Previously, radioimmunoassay (RIA) has been the only assay to measure insulin-like growth factor-1 (IGF-1) to diagnose hypersomatotropism (HS). Due to radiation concerns, availability, and the cost of IGF-1 RIA, validation of assays for automated analysers such as a chemiluminescent immunoassay (CLIA) is needed. The aim of this study was to validate a CLIA for measurement of feline IGF-1 (IMMULITE 2000^®^ XPi, Siemens Medical Solutions Diagnostics, Malvern, PA, USA) compared to IGF1 RIA, establish reference interval (RI), and determine a cut-off value for diagnosis of HS in diabetic cats. Validation of assay performance included precision, linearity, and recovery studies. Right-sided RI was determined using surplus serum of 50 healthy adult cats. Surplus serum samples of diabetic cats with known IGF-1 concentration with (*n* = 32/68) and without HS (*n* = 36/68) were used for method comparison with RIA. The cut-off for diagnosis of HS was established using receiver operating characteristic (ROC) analysis. The intra-assay coefficient of variation (CV) was ≤4.7%, and the inter-assay CV was ≤5.6% for samples with low, medium, and high IGF-1 concentration. Linearity was excellent (R^2^ > 0.99). The correlation between CLIA and RIA was very high (r_s_ = 0.97), with a mean negative bias for CLIA of 24.5%. The upper limit of RI was 670 ng/mL. ROC analysis showed an area under the curve of 0.94, with best cut-off for diagnosis of HS at 746 ng/mL (sensitivity, 84.4%; specificity, 97.2%). The performance of CLIA was good, and the RI and cut-off for HS diagnosis established in this study allow for CLIA to be used in routine work-up of diabetic cats.

## 1. Introduction

Hypersomatotropism (HS) is a common cause of feline diabetes mellitus (DM), estimated to affect approximately 17–25% of diabetic cats [1,2]. In most cases, the disease is caused by a functional somatotrophic adenoma of pars distalis of the anterior pituitary gland [3,4]. The result is excessive growth hormone (GH) secretion, without a regulating negative-feedback mechanism [5,6]. Growth hormone induces production of insulin-like growth-factor-1 (IGF-1), predominantly occurring in the liver [6]. Anabolic and catabolic effects of GH in combination with anabolic effects of IGF-1 result in the syndrome of acromegaly [6,7].

As GH is an important modulator of insulin sensitivity, insulin resistance occurs in most cats with HS [8,9]. Therefore, cats with HS commonly present with poorly controlled DM. However, several other causes of poorly controlled DM exist [10,11]. Only a quarter of cats with HS have been reported to exhibit the typical phenotypic features of acromegaly such as prognathia inferior and broadening of the face and paws [1]. Therefore, the disease might easily be missed, unless it is specifically tested for. Definitive diagnosis of HS is based on elevated GH and/or IGF-1 concentrations followed by advanced diagnostic imaging modalities, i.e., computed tomography (CT) or magnetic-resonance imaging, with the detection of an enlargement of the pituitary gland [12,13]. Measurement of GH is not commercially available, but IGF-1 concentration is reflective of GH secretion over the past 24 h and can be measured in commercial laboratories [14,15]. Furthermore, some authors suggest that measurement of IGF-1 might be a more reliable marker of GH disorders than GH because of its non-pulsatile release and less influence from exercise, stress, or food [16,17]. Therefore, measurement of IGF-1 has become a standard screening test for HS in cats [1,2,7].

Radioimmunoassay (RIA) is considered the gold-standard method for IGF-1 measurement [16]. Unfortunately, IGF-1 RIA is subject to limited availability and relatively high costs due to the use of radioactive substances. Alternative laboratory diagnostic methods, such as an enzyme-linked immunosorbent assay (ELISA) and a human chemiluminescent immunoassay (CLIA), have shown good diagnostic accuracy for measurement of feline IGF-1 [18,19,20,21]. Human assays are used due to the cross-species analogue structure of IGF-1 [14,15]. However, ELISAs are time-consuming and labour-intensive. The human IGF-1 CLIA has been run on the IMMULITE 2000^®^ (Siemens Medical Solutions Diagnostics, Malvern, PA, USA) and is thus widely available, fully automated, and cheaper [21]. However, its routine diagnostic use in cats is still hampered by the lack of reference intervals (RI) and a valid cut-off value for diagnosing HS, as these have so far been only established for the IGF-1 RIA [7]. Moreover, technology has changed as a successor model of the IMMULITE 2000^®^, the IMMULITE 2000^®^ XPi, has become available. The latter has not yet been evaluated for measurement of feline IGF-1.

The present study had several objectives. The first objective was to assess assay performance including precision, linearity, and recovery of a human CLIA run on the IMMULITE 2000^®^ XPi Immunoassay System (Siemens Medical Solutions Diagnostics, Malvern, PA, USA) for measurement of IGF-1 in cats and to perform a method comparison study to assess the accuracy of the IGF-1 CLIA compared to the reference method IFG-1 RIA. The second objective was to establish RI for IGF-1 measured by CLIA in cats. Finally, the third objective was to determine a cut-off value for the diagnosis of HS by CLIA in comparison to the reference method RIA.

## 2. Materials and Methods

### 2.1. Study Design and Samples

This study consisted of the following parts: assessment of assay performance (Section 2.3), method comparison (Section 2.4), establishment of reference interval (RI) (Section 2.5), and establishment of a cut-off value for diagnosis of HS (Section 2.6).

For the assessment of assay performance as well as for the establishment of the RI, surplus serum samples from 50 adult healthy cats presented at the Clinic for Small Animals, Justus-Liebig-University of Giessen (JLU), were used. The healthy cats’ cohort is described in more detail in Section 2.5. For method comparison and for the establishment of the cut-off value for diagnosis of HS, residual samples from 68 diabetic cats with known HS status and IGF-1 concentration previously determined by RIA submitted to the Royal Veterinary College (RVC) Companion Animal Diabetes Register or presented at the RVC Diabetic Remission Clinic were included. Diagnosis of HS in the diabetic cohort is described in more detail in Section 2.4.1.

For samples obtained at the JLU, consent of the owners was given on admission, and ethical permission to use surplus blood samples was obtained from the regional authority (Regierungspräsidium Giessen, V 54-19 c20-15 (1) Gi18/17).

For samples obtained from the RVC, ethical approval for the use of these samples for the present study was given by the Clinical Research Ethical Review Board (URN 2021 2043-3).

All samples were kept frozen at −80 °C until analysis using IGF-1 CLIA. The median storage time was 2.5 months (range 1–11 months) for samples obtained at the JLU. At the RVC, samples were stored at −80 °C for a median of 8 years (range 5–11 years) prior to shipment on dry ice to Germany, where the IGF-1 measurement with CLIA took place (see Section 2.2 below). To ensure stability of IGF-1 prior to shipment and analysis, 10 samples stored for more than 9 years (median 10, range 9–11 years) at −80 °C, with IGF-1 concentrations ranging from 896 to 1907 ng/mL, were re-analysed by RIA (see Supplementary Material S1 for details of storage times and IGF-1 concentrations). The %bias between the initial analysis and the second analysis following storage was calculated. There was a median %bias of −2.9% (range +15% to −22.2%), which was below the total allowable error (TE_a_) of 22.3% for feline IGF-1 [22,23].

Also, considering the intra-assay CV of 3.3–7.5% and inter-assay CV of 4.4–6% for RIA given by the manufacturer, and the in-house inter-assay CV of 8.5% at the laboratory performing IGF-1 measurement using RIA (personal communication, NationWide Specialist Laboratories, Pampisford, Cambridge, UK), the stability of IGF-1 was considered good.

### 2.2. Insulin-like Growth Factor-1 CLIA

Feline serum IGF-1 was measured at SYNLAB Vet using CLIA (IMMULITE^®^ 2000 XPi Immunoassay System, Siemens Medical Solutions Diagnostics, Malvern, PA, USA), a solid-phase, enzyme-labelled chemiluminescent immunometric assay, designed and validated for measurement of human IGF-1 [24]. The human IGF-1 CLIA was performed according to the manufacturer’s instructions and was run fully automated on the IMMULITE^®^ XPi analyser, requiring a sample volume of 20 μL (+100 μL additional dead space volume). The lower limit of detection (LoD) of the human IGF-1 assay was 13.3 ng/mL [25]. For samples with IGF-1 concentrations exceeding the dynamic range of the assay of 15–1000 ng/mL [25], 1:5 dilution with 0.9% NaCl was performed.

For sample measurement, the analyser worked with solid (bead pack containing test beads) and liquid phases (reagent pack), both located inside the instrument. The bead pack contained beads coated with monoclonal mouse anti-IGF-1 antibodies (capture antibody). The liquid reagent contained alkaline phosphatase (bovine calf intestine) conjugated with polyclonal rabbit anti-IGF-1 antibodies in a buffer solution (detection antibody). Sample and reagent were automatically pipetted into the test unit containing one bead. This preparation was then incubated at 37 °C with intermittent agitation. Following an incubation period of 60 min, the test unit was spun at high speed. The reaction fluid was forced up and completely captured in a collection chamber. A series of washes with diluent fluids removed unbound material from the bead and inner tube. Finally, chemiluminescent substrate was added to the test unit. Light emission was read with a sensitive photon counter, which was then converted to the corresponding IGF-1 concentration based on the extent of light emission.

All steps, including assay and quality control procedures, were performed according to the manufacturer’s instructions. Internal quality control was performed daily with two levels (normal and high) of quality control material provided by the manufacturer (Siemens Medical Solutions Diagnostics, Malvern, PA, USA) [25].

### 2.3. Assessment of Analytical Performance

#### 2.3.1. Procedures

Assessment of CLIA performance was carried out by estimation of assay precision, linearity under dilution, recovery studies, and assessment of the effect of interfering substances.

Assay precision was evaluated by assessment of within-run (intra-assay) and between-run (inter-assay) variation. Pooled surplus serum samples of expected low, medium, and high IGF-1 concentration were used to obtain sufficient sample quantity. Intra-assay precision was determined by measuring IGF-1 concentration in these three pooled samples 20 times within one analytical run on the same day. For determination of inter-assay precision, the three pooled samples were analysed daily on 20 consecutive days. For these repeated measurements, the samples were aliquoted before freezing to exclude variation due to repeated freeze–thaw cycles.

The accuracy of the assay was evaluated by assessment of linearity under dilution and recovery studies. Assessment of linearity was performed using a single serum sample with medium-to-high IGF-1 concentration (794 ng/mL). This sample was serially diluted with 0.9% NaCl to achieve specimens with a dilution factor of 1:0, 1:1, 1:3, and 1:5, respectively. Measurements were performed in triplicates.

For recovery studies, serum samples with high (847 ng/mL) and low (218 ng/mL) IGF-1 concentrations were mixed at ratios of 1:0, 9:1, 6:4, 3:7, and 0:1, and measured in triplicates.

To further investigate systematic errors, the effect of interfering substances (i.e., bilirubin, haemoglobin, and lipids) was assessed.

For assessment of impact of hyperbilirubinaemia, a stock solution with 20 g/L was prepared by adding 20 mg bilirubin (Sigma Aldrich, Saint Louis, MO, USA) to 1 mL of 0.1 molar NaOH. The influence of 0.2 g/L of bilirubin, equivalent to marked hyperbilirubinaemia, was evaluated by adding 10 μL of stock solution to 240 μL of serum.

For interference in case of haemolysis, a stock solution of lyophilised haemoglobin (Sigma Aldrich, Saint Louis, MO, USA) was prepared by dilution of 30 mg lyophilised bovine haemoglobin in 0.3 mL 0.9% NaCl. The effect of haemolysis was evaluated by mixing 20 μL of stock solution with 180 of μL serum, 10 of μL stock solution with 190 μL of serum, and 10 μL of stock solution with 390 μL of serum, to achieve concentrations of haemoglobin of 10 g/L, 5 g/L, and 2.5 g/L, respectively, reflecting marked, moderate, and mild haemolysis. For assessment of influence of hyperlipidaemia, 30 μL of undiluted lipid solution containing soybean emulsion (Intralipid 20%, Fresenius Kabi Canada, Toronto, ON, Canada) was added to 150 μL of pooled serum to achieve a lipid concentration of 30 g/L, corresponding to marked hyperlipidaemia.

A pooled surplus serum sample with a low IGF-1 concentration of approximately 360 ng/mL was used in aliquots for each potentially interfering substance. The remaining aliquot was used as a control sample by spiking with the same volume of diluent used for preparation of the stock solution of the interfering substance, i.e., either 0.9% NaCl (in case of haemoglobin) or NaOH (in case of bilirubin). The lipid solution was used for spiking without dilution. Measurements were performed in triplicates.

#### 2.3.2. Performance Goals and Statistical Analysis

Performance goals were set according to the guidelines of the American Society of Veterinary Clinical Pathology (ASVCP) [22]. A total allowable error (TE_a_) of 24% has been described for human IGF-1 [26]. In veterinary medicine, only one study using an ELISA established a TE_a_ for feline IGF-1 of 22.3% [23]. This quality criterion was also used in the present study. In addition, guidelines on bioanalytical method validation of the European Medicines Agency (EMA) were used for assessment of acceptable performance (CV < 20% = CV_20%_) [27].

For estimation of assay precision, mean, standard deviation (SD), as well as coefficient of variation (CV) were calculated:

$$CV\%=\frac{SD}{mean}\times 100$$

Linearity under dilution was evaluated by computing mean, SD, CV, and ordinary linear regression analysis with estimation of coefficient of determination (R^2^) for comparison of observed and expected IGF-1 concentrations.

Percentage of recovery was calculated:

$$Recovery\%=\frac{measured\;concentration}{expected\;concentration}\times 100$$

The accuracy of the assay was considered acceptable if the percentage of recovery ranged between 80 and 120% [27,28]. Inaccuracy (bias) in percentage between expected and measured mean IGF-1 concentration was calculated:

$$bias\;in\%=\frac{mean\;target-mean\;measured}{mean\;target}$$

Additionally, observed total error (TE_obs_) was calculated and compared to quality goal TE_a_ of 22.3%.

For interference studies, mean IGF-1 concentrations of interferent-containing sample and unaltered controls were compared. The criterion for acceptable performance was bias as systematic error (expressed as difference between means) < TE_a_ of 22.3%.

Statistical analysis was performed using commercially available software (GraphPad Prism 6, Graph Pad software Inc., Boston, MA, USA; MedCalc Version 20.019, MedCalc software Ltd., Ostend, Belgium and Microsoft Excel Version 16.631.1). The level of significance was set at *p* < 0.05.

### 2.4. Method Comparison RIA and CLIA

#### 2.4.1. Diagnosis of HS

For method comparison, surplus serum samples of 68 diabetic cats with known IGF-1 concentration, previously measured by RIA (Mediagnost IGF-R20, Mediagnost, Reutlingen, Germany, at the NationWide Specialist Laboratories, Pampisford, Cambridge, UK), were used. In all cats, IGF-1 was measured after a minimum of 6 weeks of insulin treatment [29]. Residual serum samples from cats diagnosed with DM with and without HS were included. Diagnosis of HS was made if any of the following criteria were satisfied:

- Single IGF-1 concentration > 1000 ng/mL (measured by RIA) [7] and an enlarged pituitary on CT (*n* = 20);
- Repeatedly increased IGF-1 concentration > 1000 ng/mL in combination with clinical evidence of insulin resistance (poorly controlled DM despite insulin dose > 1 U/kg BID) in cats, where diagnostic imaging was not available (*n* = 7);
- IGF-1 concentration < 1000 ng/mL in combination with clinical evidence of insulin resistance and enlarged pituitary on CT (*n* = 2) or, if imaging was not available, IGF-1 > 1000 ng/mL reported at later timepoint (i.e., sample with IGF-1 < 1000 ng/mL was used for this study, but the cat had a later IGF-1 measurement > 1000 ng/mL) (*n* = 3).

All but one diabetic cat without HS had IGF-1 < 1000 ng/mL measured by RIA. Hypersomatotropism was excluded in these cats based on no evidence of insulin resistance and good diabetic control or diabetic remission during a follow-up period of a minimum of 1 year (*n* = 36). Computed tomography of the head was only available in 4 diabetic cats without HS, all with normal-sized pituitaries. This included the single diabetic cat with IGF-1 > 1000 ng/mL, considered not to have HS based on the CT findings and good diabetic control, with no clinical evidence of insulin resistance.

#### 2.4.2. Statistical Analysis

IGF-1 values previously measured by reference method RIA in 68 diabetic cats with (*n* = 32) and without HS (*n* = 36) were compared to CLIA measurements performed in the present study. The IGF-1 concentration in these samples previously determined by RIA ranged from 85 to 1907 ng/mL.

Statistical analysis was performed using the software MedCalc Version 20.019 (MedCalc Software Ltd., Ostend, Belgium). Spearman’s rank correlation coefficient (r_s_) was calculated to assess association between methods because of non-parametric distribution of IGF-1 values. The strength of association was considered very high for r_s_ between 0.9 and 1.0, high for r_s_ between 0.7 and 0.89, moderate for r_s_ between 0.5 and 0.69, low for r_s_ between 0.3 and 0.49, and little if any for r_s_ < 0.29 [30].

To assess if systemic or proportional difference exists between CLIA and RIA, Passing–Bablok regression analysis was performed. The intercept A (i.e., measure of systematic difference between methods) and slope B (i.e., proportional difference between methods) were calculated with their 95% confidence intervals (CIs) [31,32].

For calculation of mean difference (bias in %) between CLIA and RIA, Bland–Altman analysis was performed by plotting differences as % between the two methods (CLIA and RIA) against the averages of the two methods. The limits of agreement (LoA) were defined as the mean difference ± 1.96 SD of differences.

### 2.5. Establishment of Reference Interval

For establishment of RI for IGF-1 CLIA, surplus serum samples of 50 client-owned cats presented at the JLU for health examinations or blood donations between August 2020 and June 2021 were used.

All cats were considered healthy based on complete history, physical examination, complete blood count, and clinical biochemistry. Only adult cats between 12 months and 11 years of age were included. Blood was collected by cephalic or jugular venipuncture, placed into plain serum collection tubes (micro collection tube, 1.3 mL, SARSTEDT AG & Co., Nümbrecht, Germany), and separated by centrifugation at 18,620 G for one minute within 30–60 min of collection.

To establish RI for IGF-1 CLIA, the data were assessed for normality by visual inspection of the histogram and Q–Q plot as well as the Shapiro–Wilk test using Excel-based freeware software Reference Value Advisor version 2.1 [33]. Detection of potential outliers was carried out with the Tukey’s and Dixon’s tests. The reference interval was calculated using MedCalc Version 20.019, MedCalc software Ltd., Ostend, Belgium. Due to non-normal data distribution, right-sided 95% RI was calculated using a robust method after Box–Cox transformation, specifying the 90% CI as recommended by ASVCP guidelines [22]. A potential impact of age on IGF-1 concentration was assessed with weighted polynomial regression analysis.

### 2.6. Cut-Off for Diagnosis of HS via CLIA

For establishment of the cut-off for IGF-1 CLIA to detect HS, IGF-1 concentrations measured by CLIA of the above-mentioned 68 diabetic cats with known HS status (32 with HS and 36 without) were used (see Section 2.4.1 for details on HS diagnosis).

Receiver operating characteristic (ROC) analysis was performed using MedCalc Version 20.019 (MedCalc software Ltd., Ostend, Belgium) to assess the ability of IGF-1 measured by CLIA to differentiate between diabetic cats with and without HS, and to establish the optimal cut-off for diagnosis of HS (chosen to achieve the maximum sensitivity and specificity). Besides the best cut-off for diagnosis of HS, the grey area of IGF-1 values was defined as the range between the cut-off with the sensitivity of 100% and the cut-off with the specificity of 100%.

## 3. Results

### 3.1. Assessment of Analytical Performance

#### 3.1.1. Precision of CLIA

The intra-assay CV was 2.5%, 4.7%, and 1.6%, and the inter-assay CV was 5.1%, 5.6%, and 4.1% for a sample with low, medium, and high IGF-1 concentration, respectively. All CVs fulfilled the quality criterion of TE_a_ < 22.3% and the EMA guidelines threshold of <20% (Table 1).

#### 3.1.2. Linearity of CLIA

**Figure 1:**
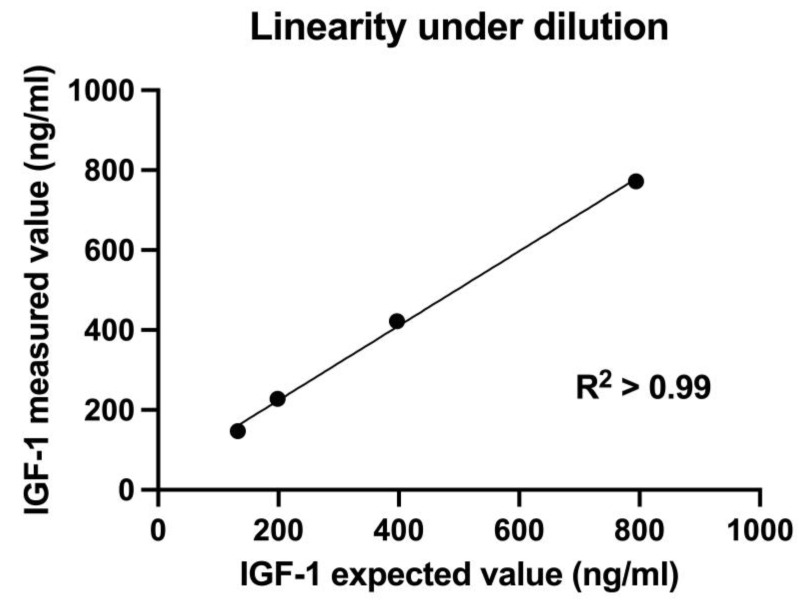
Linearity under dilution of a serum sample with medium-to-high insulin-like growth factor-1 (IGF-1) concentration (794 ng/mL) measured with chemiluminescent immunoassay. Linear regression analysis showed coefficient of determination R^2^ > 0.99, indicating an excellent linearity under dilution.

Linearity under serial dilution of a serum sample with medium-to-high IGF-1 concentration (794 ng/mL) was demonstrated with a coefficient of determination (R^2^) of 0.99 (Figure 1).

Based on the ratio of the measured and expected concentrations in the linearity study, recovery ranged from 97.2% to 114.9%, with a mean of 107.4% (Table 2).

Recovery after mixing samples with high (874 ng/mL) and low (218 ng/mL) IGF-1 concentration ranged between 98.6% and 103.8%, with a mean of 101.4% (Table 3).

All recovery rates were within the acceptable range of 80–120%. Bias was below the desired TE_a_ of 22.3%.

#### 3.1.3. Interference Study

**Figure 2:**
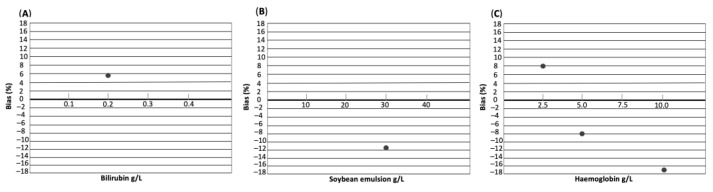
Interferograph depicting the effect of interfering substances bilirubin (**A**), soybean emulsion (**B**), and haemoglobin (**C**) on IGF-1 concentration. The concentration of each interfering substance in g/L is shown on the x-axis. Bias in % is shown on the y-axis.

Based on %bias < TE_a_, none of the interfering substances had a substantial effect on IGF-1 concentration measured by CLIA (Table 4 and Figure 2). However, an increase in %bias could be observed with increasing haemoglobin concentration.

### 3.2. Method Comparison RIA and CLIA

**Figure 3:**
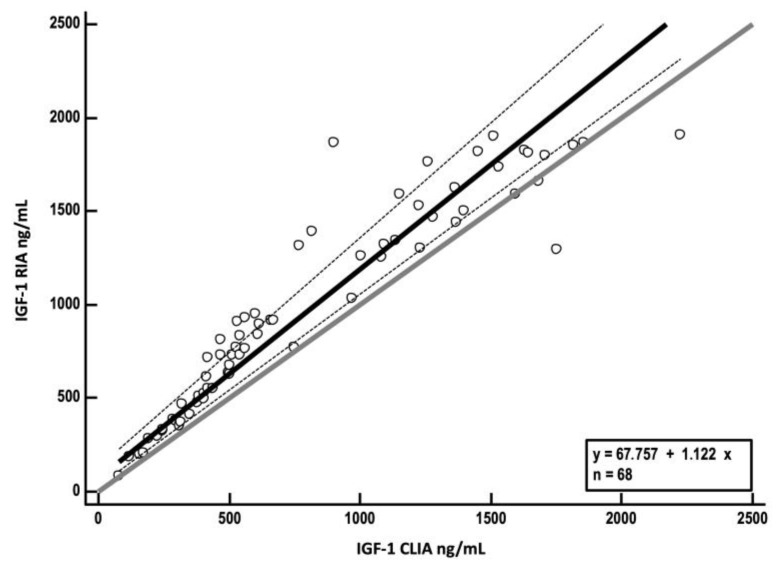
Passing–Bablok regression analysis of insulin-like growth factor-1 (IGF-1) concentrations measured by radioimmunoassay (RIA) (y-axis) plotted against values obtained by chemiluminescent immunoassay (CLIA) (x-axis). The solid black line represents the line of best fit derived from regression analysis (regression line); dashed lines show 95% confidence interval for the regression line. The solid grey line represents the identity line (x = y).

Spearman’s rank correlation coefficient of r_s_ = 0.97 (*p* < 0.0001) indicated a very high correlation between methods. Passing–Bablok regression analysis yielded an intercept A of 67.8 (95% CI, 25.7–130.6) and a slope B of 1.1 (95% CI, 1–1.2), indicating a constant, but not proportional, bias between CLIA and RIA (Figure 3).

**Figure 4:**
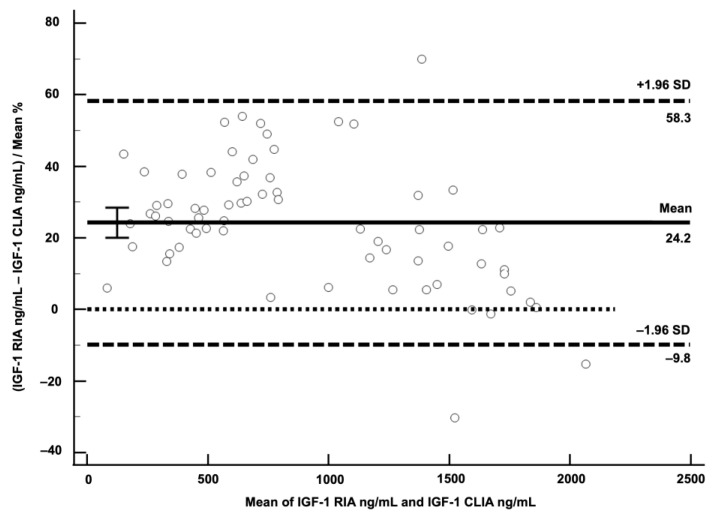
Bland–Altman plot for visualisation of difference between chemiluminescent immunoassay (CLIA) and radioimmunoassay (RIA). Differences of the two methods expressed as % were plotted against average of IGF-1 concentrations of the two methods. Solid horizontal line represents mean difference of both methods in %, indicating negative bias of 24.2% for CLIA. Dashed horizontal lines represent limits of agreement. The dotted horizontal line is the zero line.

A Bland–Altman plot showed a mean negative bias for CLIA of 24.2% (SD 17.4) with LoA from −9.8 to 58.3% (Figure 4).

A bias of 24.2% was higher than feline TE_a_ < 22.3%, and failed the human TE_a_ of <24%, indicating that CLIA and RIA cannot be used interchangeably.

### 3.3. Establishment of Reference Interval

**Figure 5:**
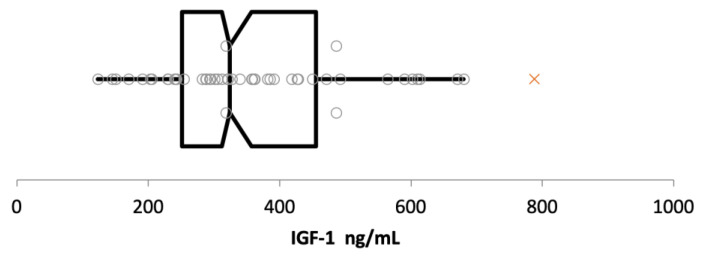
Box-and-whisker plot of insulin-like growth factor-1 (IGF-1) concentrations measured by chemiluminescent immunoassay in ng/mL. The left and the right sides of the box are the lower and upper quartiles (i.e., 25–75%), the vertical line splitting the box into two represents the median IGF-1 concentration of 324 ng/mL. Whiskers represent the minimum (left whisker, IGF-1 concentration of 123 ng/mL) and maximum (right whisker, IGF-1 concentration of 680 ng/mL) values. Each grey circle represents an IGF-1 value of a single cat; a single suspect outlier detected by Tukey’s test (IGF-1 concentration of 788 ng/mL) is represented by a red cross.

Fifty cats, with a median age of 5 years (range: 1–11), were included to establish the RI. Of these 50 cats, 27/50 were neutered males, 21/50 were spayed females, and 2/50 were intact females. There were 30 Domestic Shorthair, five Maine Coon, four British Shorthair, three Bengal, two Ragdoll, and one each of Siberian, Scottish Fold, Norwegian Forest Cat, Birman Cat, Chartreux, and Neva Masquerade. The right-sided RI was 670 ng/mL (90% CI 575 to 761) (Figure 5).

There was a single suspect outlier with an IGF-1 concentration of 788 ng/mL, which was the IGF-1 concentration in a moderately obese 5-year-old Norwegian Forest Cat, with a body weight of 6.9 kg and a body condition score of 7/9. A repeated measurement of the IGF-1 concentration of this cat about 6 months later again revealed a high value (915 ng/mL) in an otherwise clinically healthy cat, without any evidence of cardiac or endocrine disease or any clinicopathological abnormalities.

For cats aged between 1 and 11 years, there was no impact of age on the IGF-1 concentration (*p* = 0.30).

### 3.4. Cut-Off CLIA for the Diagnosis of HS in Diabetic Cats

**Figure 6:**
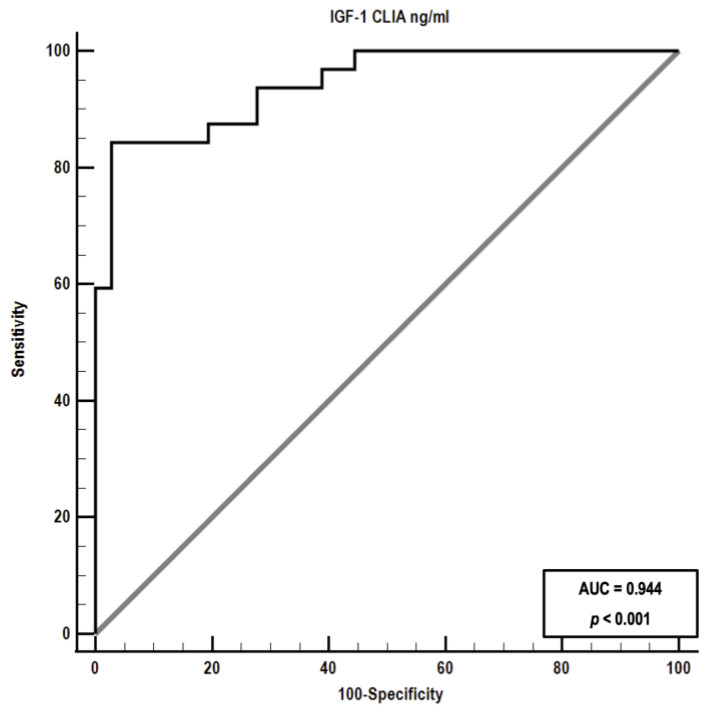
Receiver operating characteristic (ROC) curve depicting the ability of insulin-like growth factor-1 (IGF-1) measured by chemiluminescent immunoassay to differentiate between diabetic cats with and without hypersomatotropism. The area under the curve (AUC) was 0.94.

Receiver operating characteristic analysis revealed an area under the curve (AUC) of 0.94 (*p* < 0.001) for IGF-1 measured by CLIA, indicating that this parameter can differentiate between diabetic cats with and without HS (Figure 6).

**Figure 7:**
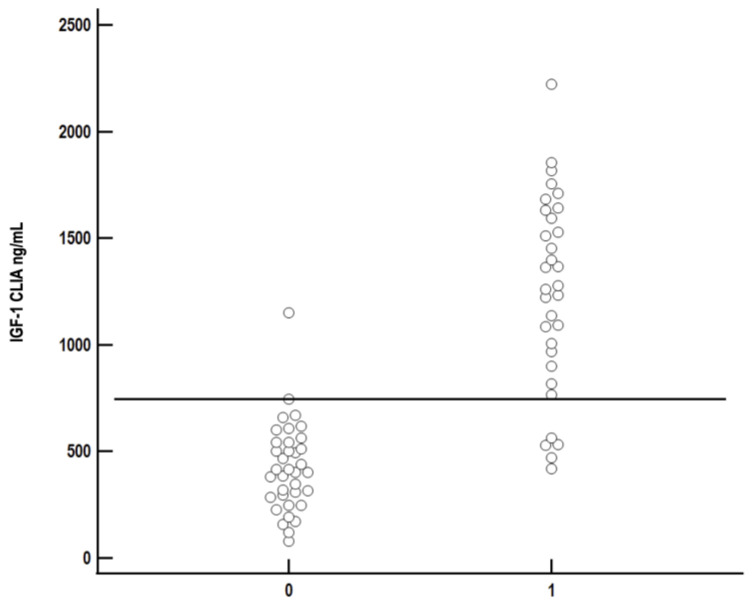
Comparison of serum insulin-like growth factor-1 (IGF-1) concentration measured by chemiluminescent immunoassay (CLIA) between diabetic cats with (1) and without (0) hypersomatotropism. Each circle represents IGF-1 concentration of a single cat. The best cut-off to differentiate between diabetic cats with and without HS (sensitivity 84.4%, specificity 97.2%) was 746 ng/mL and is represented as the solid black line.

The best cut-off for IGF-1 measured by CLIA was 746 ng/mL, having a sensitivity of 84.4% and specificity of 97.2% for diagnosis of HS (Figure 7).

The grey area for IGF-1 concentration was between 400 ng/mL (sensitivity 100%, specificity 44.4%) and 1175 ng/mL (sensitivity 59.4%, specificity 100%).

## 4. Discussion

This study validated a commercially available human IGF-1 CLIA (IMMULITE^®^ 2000 XPi Immunoassay System) for measurement of feline IGF-1 in serum samples. Overall, an excellent analytical performance in terms of precision and accuracy of the IGF-1 CLIA was demonstrated. Within-run precision was excellent for all three IGF-1 concentrations (CVs ≤ 4.7%) and was comparable with data provided by the manufacturer for human samples, with CVs of 2.9%, 3.0%, and 2.3% for samples with low, medium, and high IGF-1 concentration, respectively [25]. Inter-assay CVs were lower in this feline study (CVs ≤ 5.6%) when compared to human samples (CVs ≤ 7.6%) [25]. All CVs for feline samples fulfilled performance criteria proposed by EMA (CV < 20%) [27] and TE_a_ for feline IGF-1 of 22.3% identified by a previous study [23].

Accuracy was also excellent for CLIA, fulfilling acceptability criterion with TE_obs_ < TE_a_ in both linearity and recovery studies [22,26,32]. Furthermore, similar to data provided by the assay manufacturer for human IGF-1, there was no significant effect of interfering substances on measurement of feline IGF-1, despite testing higher concentrations of interfering substances than previously performed for humans (bilirubin at a concentration of 0.2 g/L and haemoglobin at a concentration of 5 g/L were tested in humans) [25]. However, it should be noted that the presence of marked haemolysis (haemoglobin concentration of 10 g/L) increased the %bias to 17% in comparison to mild-to-moderate haemolysis, with a %bias of 8%. Insulin-like growth factor-1 concentration in the sample with marked haemolysis was lower than in the non-haemolytic control, which might be clinically relevant when haemolytic samples with IGF-1 concentration at the upper limit of RI or those with IGF-1 concentration around the cut-off for HS (see below) are analysed. Potentially, diagnosis of HS could be missed in such cases. For this reason, assessment of the effect of haemolysis on IGF-1 using samples with higher IGF-1 concentrations (a sample with low concentration was used in the present report) and by spiking samples with a higher haemoglobin concentration than in this investigation might be warranted in future studies.

The right-sided RI for IGF-1 measured by CLIA established in this study was 670 ng/mL. Due to differences in methodology, it is not possible to directly compare RI established by different assays. Only two studies using CLIA were identified by a literature search. A non-peer-reviewed student thesis measured IGF-1 in 92 feline plasma samples using the same CLIA (IMMULITE 2000 XPi) and established a right-sided RI of 795 ng/mL [34]. However, on careful reading the thesis, it was unclear if the cats included in the study were healthy, because samples from laboratory submissions without knowledge of medical history were used. Therefore, the RI established in that previous study is questionable. In another investigation, Tschuor et al. compared different assays for measurement of IGF-1 in healthy and diseased cats [21]. Among others, CLIA was evaluated and compared with the RIA that was also used as the gold standard in our study. Although RI was not calculated, the median IGF-1 of 279 ng/mL (range 12.5–525) in 39 healthy cats measured by CLIA in that study was lower than the median IGF-1 concentration of 324 ng/mL (range 123–788) found by us. We did not find any impact of age on IGF-1 concentration; however, interestingly, Tschuor et al. reported significantly higher IGF-1 concentrations in 19 young cats (median age 3 years) in comparison to 20 middle-aged to older cats (median age 10 years) when IGF-1 was measured by CLIA but not when it was measured by RIA. The median age of cats included in our investigation was 5 years, which could potentially be one of the factors explaining higher RI identified in this study when compared to the range of IGF-1 concentrations identified in healthy cats by Tschuor et al. [21]. In human medicine, age-specific reference ranges for paediatric, adult, and geriatric patients exist for IGF-1 [25,35]. Because the cats used for establishment of RI in our study were young or mature adults [36], the effect of older age on IGF-1 concentration could not be evaluated here. Future studies are needed to assess the effect of age on IGF-1 concentration in cats covering a wider age interval than in our study.

Another factor potentially affecting IGF-1 concentration and impacting the RI might be the body weight of included cats. Although weight gain or weight loss was not associated with significant changes in IGF-1 concentration in a small study including ten healthy research cats [37], Strage et al. detected a significant association between body weight and IGF-1 in 55 healthy cats [23]. In that previous study, an increase of 1 kg in body weight was associated with an estimated increase in IGF-1 concentration of 38% [23]. Unfortunately, body weight was documented only in some cats used to establish the RI in the present study, preventing assessment of the effect of body weight on IGF-1. The effect of body weight was not assessed in Tschuor et al.’s study either [21].

Body weight is also closely associated with breed, but to the authors’ knowledge, breed-specific RIs for IGF-1 have not been reported in cats. In the present study, cats of large (four British Shorthairs, two Ragdolls, one Norwegian Forest Cat, and one Siberian) and giant (five Maine Coons) breeds [38] were included, but no data on the breed(s) of the included cats were provided by Tschuor et al. [21]. Therefore, if differences in included breeds have contributed to differences in IGF-1 values in healthy cats in this and the previous study, [21] cannot be evaluated. Unfortunately, the number of cats of large-to-giant breeds included in our study was too small to reliably assess the impact of breed size on IGF-1 concentration. However, given the impact of body weight on IGF-1 concentrations identified by Strage at al. [23], the establishment of breed-specific RIs should be considered in future studies. Indeed, the suspect outlier with IGF-1 concentration of 788 ng/mL was a Norwegian Forest Cat, that is considered to be a large cat breed [38].

Since IGF-1 measurement in cats is mainly used to diagnose HS, only higher concentrations are clinically relevant, which is why a one-sided RI was chosen in this study. Hyposomatotropism or pituitary dwarfism is a very rare condition in cats, where IGF-1 concentrations are expected to be low or undetectable [39,40]. For appropriate interpretation of the IGF-1 values in suspected cases, comparison with concentrations of healthy littermates is recommended [39,40]. Low IGF-1 concentrations might also occur in cats newly diagnosed with DM as well as in cats with lymphoma and, depending on the assay, also in aging animals [21,23,29]. These conditions might need to be considered when interpreting IGF-1 measurements. In newly diagnosed diabetic cats, repeated measurements are advised, or IGF-1 should at least be measured after several weeks of insulin treatment [29,41]. On the other hand, increased IGF-1 concentration might not be specific for HS. A recent study revealed increased IGF-1 concentrations in some non-diabetic cats diagnosed with hypertrophic cardiomyopathy (HCM) [42]. Whether those cats suffered from HS could not be evaluated in that study because IGF-1 was measured retrospectively in stored blood samples from cats diagnosed with HCM, and cranial imaging was not performed. These cats were not diabetic, and, as discussed by the authors, the use of IGF-1 cut-off determined in a diabetic population might not be appropriate in non-diabetic cats [36]. The prevalence of HS in non-diabetic cats is unknown, with only few cases reported [43,44], making establishment of any cut-off for HS diagnosis in non-diabetic cats extremely difficult [43,44]. In the present study, only diabetic cats were investigated.

Although RIA is the gold standard for IGF-1 measurement, this assay is costly and not widely available due to legal restrictions regarding work with radioactive substances. Therefore, validation of automated assays such as CLIA presented in this study is needed, to overcome these issues [20]. Although a good correlation with RIA was demonstrated for CLIA in the present study, the Passing–Bablok analysis indicated that there is a constant difference between the two methods, with a negative bias of 24.2% for CLIA identified by Bland–Altmann analysis. This bias corresponds roughly to the data of Tschuor et al., who found a difference between medians of CLIA and RIA concentration of about 21% [21]. These results suggest that the two methods are not interchangeable, and correspondingly lower values of IGF-1 are measured by CLIA when compared to RIA, due to the bias. Therefore, a new cut-off for diagnosis of HS for CLIA was calculated, using IGF-1 values of 68 diabetic cats with (*n* = 32) and without (*n* = 36) HS. As expected, the IGF-1 cut-off for diagnosis of HS measured by CLIA is lower (746 ng/mL) than the RIA cut-off of 1000 ng/mL used in previous studies [1,7]. Knowing these differences between IGF-1 RIA and CLIA, it is important that follow-up samples are sent to the same laboratory, or at least a laboratory using the same assay. Furthermore, although a positive predictive value of 95% for IGF-1 > 1000 ng/mL measured by RIA was reported in a large study including 1221 diabetic cats (319 had IGF-1 > 1000 ng/mL, 63/319 had cranial imaging or necropsy, and HS was confirmed in 60/63) [1], ideally, results of cranial imaging and clinical suspicion (e.g., typical phenotypic features and evidence of insulin resistance) should be considered when making a diagnosis of HS.

This study had some limitations. The main limitation is the extended storage time of samples from diabetic cats used to compare CLIA with RIA and establish cut-off for HS. Because the storage time of IGF-1 samples previously measured by RIA ranged between 5 and 11 years, IGF-1 concentration was re-measured by RIA in ten samples with long-term storage, to assure stability. Although the stability of IGF-1 in those ten samples could be confirmed, ideally, all 68 samples would have been re-measured by RIA prior to CLIA measurement. Unfortunately, this was not possible given the high costs of the RIA. As all samples were stored at the same facility (Royal Veterinary College) with temperature monitoring equipped with alarms for all freezers, degradation of IGF-1 in the remaining samples was considered unlikely but could not be completely ruled out. Another limitation is that cranial imaging was not performed in all cats with HS to confirm the diagnosis, and only four cats without HS had a head CT. However, these were all samples from clinical patients, where diagnostic tests can only be performed when indicated (i.e., there is no indication for head CT in well-controlled diabetic cats or those that even achieved remission of their DM). Additionally, most cats had a long follow-up and were treated for their DM (or HS) at a specialised clinic (Diabetic Remission Clinic at the RVC) by board-certified veterinary internists with ample experience in treating diabetic cats, making misclassification of cases in terms of having or not having HS unlikely. Furthermore, because only diabetic cats with and without HS were included, the IFG-1 cut-off for diagnosis of HS established in this study might not be applicable to non-diabetic cats. As already discussed, only rare reports of cats with HS but without DM exist; therefore, establishing IGF-1 cut-off for diagnosis of HS in non-diabetic cats is challenging and barely possible. Future studies are also needed to assess the effect of age, body weight and breed on IGF-1 concentration measured by CLIA, because the present study either lacked the necessary data (body weight), did not include cats older than 10 years to be compared with younger cats, or did not include sufficient numbers of cats per breed to establish breed-specific RIs. Another limitation is that TE_a_ used to assess IGF-1 CLIA performance were adopted from a study that used an ELISA rather than a CLIA. That study calculated a TE_a_ of 22.3% [23]. This is similar to the cut-off of 24% reported for human IGF-1 in the Westgard Desirable Biological Variation database, without specification of reference [26]. The CLIA validated in the present study met the required specifications.

On a final note, laboratories using the CLIA assay, need to be aware of the dynamic range of the assay, which lies between 15 and 1000 ng/mL. A number of cats with HS might have IGF-1 values higher than 1000 ng/mL, making sample dilution necessary.

## 5. Conclusions

The current study demonstrates that measurement of serum IGF-1 in cats using commercially available CLIA is accurate and precise. Thus, IGF-1 measurement by CLIA can serve as a good alternative to RIA as a screening test for HS in diabetic cats. However, IGF-1 concentrations measured by CLIA and RIA are not directly comparable, and using assay-specific, and ideally laboratory-specific, RIs and cut-offs for HS is essential.

Compared to RIA, CLIA does not require radioactivity, is less expensive, and can be performed on large-scale commercial equipment available in larger veterinary laboratories. Therefore, it can be made available to a larger patient population in routine diagnostics. A possibly underdiagnosed HS can thus be detected more easily, which may contribute to better understanding and treating of unstable diabetic cats.

## Figures and Tables

**Table 1:** Intra-assay precision of a chemiluminescent immunoassay for measurement of insulin-like growth factor-1 (IGF-1) determined with 20 consecutive measurements of pooled feline serum samples with low, medium, and high IGF-1 concentrations within one analytical run, as well as inter-assay precision determined by measuring IGF-1 in samples with low, medium, and high concentrations on 20 consecutive days.

Precision	IGF-1 Sample	Mean (ng/mL)	SD (ng/mL)	CV %	CV < CV_20%_ CV < TE_a_
Intra-Assay	Low	250.2	6.3	2.5	Yes
	Medium	731.8	34.1	4.7	Yes
	High	881.7	14.5	1.6	Yes
Inter-Assay	Low	250.4	12.7	5.1	Yes
	Medium	745	41.4	5.6	Yes
	High	881	36.3	4.1	Yes

Abbreviations: SD = standard deviation, CV = coefficient of variation, TE_a_ = total allowable error (TE_a_ < 22.3%), CV_20%_ (CV < 20%).

**Table 2:** Recovery rates for insulin-like growth factor-1 (IGF-1) measured with chemiluminescent immunoassay. A serially diluted serum sample with medium-to-high IGF-1 concentration (794 ng/mL) was measured in triplicates.

Dilution Factor	Expected Value (ng/mL)	Mean Measured Value (±SD) (ng/mL)	CV%	%CV < CV_20%_ CV < TE_a_	Recovery %	Bias	TE_obs_	TE_obs_ < TE_a_
1:0	794	772 (±28.4)	3.7	Yes	97.2	−2.8	10.2	Yes
1:1	397	422 (±6.6)	1.6	Yes	106.3	6.3	9.5	Yes
1:3	198.5	228.3 (±6.5)	2.9	Yes	114.9	15	20.8	Yes
1:5	132.3	147.3 (±2.5)	1.7	Yes	111.3	11.3	14.7	Yes

Abbreviations: CV = coefficient of variation, SD = standard deviation, TE_obs_ = observed total error, TE_a_ = total allowable error (TE_a_ < 22.3%), CV_20%_ (CV < 20%).

**Table 3:** Percentage recovery for insulin-like growth factor-1 (IGF-1) measured with chemiluminescent immunoassay after mixing a serum sample of high (874 ng/mL) and low (218 ng/mL) IGF-1 concentration at different ratios, measured in triplicates.

Volume % High IGF-1 Sample	Volume % Low IGF-1 Sample	Expected Value (ng/mL)	Mean Measured Value (±SD) (ng/mL)	CV %	%CV < CV_20%_ %CV < TE_a_	Recovery %	Bias	TE_obs_	TE_obs_ < TE_a_
100	0	874	888 (±18.5)	2.1	Yes	101.6	1.6	5.8	Yes
90	10	808.4	821.7 (±32.6)	4	Yes	101.6	1.6	9.6	Yes
60	40	611.6	603 (±13)	2.2	Yes	98.6	−1.4	5.8	Yes
30	70	414.8	421.3 (±7.2)	1.7	Yes	101.6	1.6	5	Yes
0	100	218	226.3 (±7.4)	3.3	Yes	103.8	3.8	10.4	Yes

Abbreviations: CV = coefficient of variation, SD = standard deviation, TE_obs_ = observed total error, TE_a_ = total allowable error (TE_a_ < 22.3%), CV_20%_ (CV < 20%).

**Table 4:** Assessment of the effect of interfering substances (bilirubin, lipids, and haemoglobin) on insulin-like growth factor-1 (IGF-1) concentration measured by chemiluminescent immunoassay. The effect of interfering substances was considered negligible.

Interferent Concentration	Mean IGF-1_control_ (ng/mL) ± SD	Mean IGF-1_test_ (ng/mL) ± SD	Bias (ng/mL)	%Bias	%Bias < TE_a_
Bilirubin 0.2 g/L	330 ± 12.9	349 ± 7.9	19	5.8	Yes
Soybean emulsion 30 g/L	361 ± 14.2	319.7 ± 19.6	−41.3	−11.4	Yes
Haemoglobin 2.5 g/L	326 ± 18.3	352 ± 14	26	8	Yes
Haemoglobin 5 g/L	326 ± 18.3	300 ± 9.6	−26	−8	Yes
Haemoglobin 10 g/L	326 ± 18.3	270.7 ± 12.3	−55.3	−17	Yes

Abbreviations: TE_a_ = total allowable error (TE_a_ < 22.3%), SD = standard deviation.

## Data Availability

The data presented in this study are available on request from the corresponding author.

## Supplementary Materials

The following supporting information can be downloaded at: https://www.mdpi.com/article/10.3390/vetsci10090575/s1, Table S1: Results of first and repeated 10 IGF-1 samples measured by radioimmunoassay stored >9 years at −80 °C.

## References

[1] Niessen S.J.M., Forcada Y., Mantis P., Lamb C.R., Harrington N., Fowkes R., Korbonits M., Smith K., Church D.B. (2015). Studying Cat (*Felis catus*) Diabetes: Beware of the Acromegalic Imposter. PLoS ONE.

[2] Schaefer S., Kooistra H.S., Riond B., Suchodolski J.S., Steiner J.M., Prins M., Zini E., Reusch C.E. (2017). Evaluation of Insulin-like Growth Factor-1, Total Thyroxine, Feline Pancreas-Specific Lipase and Urinary Corticoid-to-Creatinine Ratio in Cats with Diabetes Mellitus in Switzerland and the Netherlands. J. Feline Med. Surg..

[3] Van Bokhorst K.L., Galac S., Kooistra H.S., Valtolina C., Fracassi F., Rosenberg D., Meij B.P. (2021). Evaluation of Hypophysectomy for Treatment of Hypersomatotropism in 25 Cats. J. Vet. Intern. Med..

[4] Fenn J., Kenny P.J., Scudder C.J., Hazuchova K., Gostelow R., Fowkes R.C., Forcada Y., Church D.B., Niessen S.J.M. (2021). Efficacy of Hypophysectomy for the Treatment of Hypersomatotropism-induced Diabetes Mellitus in 68 Cats. J. Vet. Intern. Med..

[5] Barkan A.L., Stred S.E., Reno K., Markovs M., Hopwood N.J., Kelch R.P., Beitins I.Z. (1989). Increased Growth Hormone Pulse Frequency in Acromegaly*. J. Clin. Endocrinol. Metab..

[6] Casanueva F.F. (1992). Physiology of Growth Hormone Secretion and Action. Endocrinol. Metab. Clin. N. Am..

[7] Niessen S.J.M., Petrie G., Gaudiano F., Khalid M., Smyth J.B.A., Mahoney P., Church D.B. (2007). Feline Acromegaly: An Underdiagnosed Endocrinopathy?. J. Vet. Intern. Med..

[8] Scudder C.J., Gostelow R., Forcada Y., Schmid H.A., Church D., Niessen S.J.M. (2015). Pasireotide for the Medical Management of Feline Hypersomatotropism. J. Vet. Intern. Med..

[9] Gostelow R., Scudder C., Keyte S., Forcada Y., Fowkes R.C., Schmid H.A., Church D.B., Niessen S.J.M. (2017). Pasireotide Long-Acting Release Treatment for Diabetic Cats with Underlying Hypersomatotropism. J. Vet. Intern. Med..

[10] Niessen S.J.M., Church D.B., Forcada Y. (2013). Hypersomatotropism, Acromegaly, and Hyperadrenocorticism and Feline Diabetes Mellitus. Vet. Clin. N. Am. Small Anim. Pract..

[11] Caney S.M.A. (2013). Pancreatitis and Diabetes in Cats. Vet. Clin. N. Am. Small Anim. Pract..

[12] Posch B., Dobson J., Herrtage M. (2011). Magnetic Resonance Imaging Findings in 15 Acromegalic Cats. Vet. Radiol. Ultrasound.

[13] Lamb C.R., Ciasca T.C., Mantis P., Forcada Y., Potter M., Church D.B., Niessen S.J. (2014). Computed Tomographic Signs of Acromegaly in 68 Diabetic Cats with Hypersomatotropism. J. Feline Med. Surg..

[14] Lewitt M., Hazel S., Church D., Watson A., Powell S., Tan K. (2000). Regulation of Insulin-like Growth Factor-Binding Protein-3 Ternary Complex in Feline Diabetes Mellitus. J. Endocrinol..

[15] Starkey S.R., Tan K., Church D.B. (2004). Investigation of Serum IGF-I Levels amongst Diabetic and Non-Diabetic Cats. J. Feline Med. Surg..

[16] Niessen S.J.M., Khalid M., Petrie G., Church D.B. (2007). Validation and Application of a Radioimmunoassay for Ovine Growth Hormone in the Diagnosis of Acromegaly in Cats. Vet. Rec..

[17] Scott-Moncrieff J.C. (2010). Insulin Resistance in Cats. Vet. Clin. N. Am. Small Anim. Pract..

[18] Tvarijonaviciute A., German A.J., Martínez-Subiela S., Tecles F., Ceron J.J. (2012). Analytical Performance of Commercially-Available Assays for Feline Insulin-like Growth Factor 1 (IGF-1), Adiponectin and Ghrelin Measurements. J. Feline Med. Surg..

[19] Frystyk J., Freda P., Clemmons D.R. (2010). The Current Status of IGF-I Assays—A 2009 Update. Growth Horm. IGF Res..

[20] Rosca M., Forcada Y., Solcan G., Church D.B., Niessen S.J.M. (2014). Screening Diabetic Cats for Hypersomatotropism: Performance of an Enzyme-Linked Immunosorbent Assay for Insulin-like Growth Factor 1. J. Feline Med. Surg..

[21] Tschuor F., Zini E., Schellenberg S., Wenger M., Boretti F.S., Reusch C.E. (2012). Evaluation of Four Methods Used to Measure Plasma Insulin-like Growth Factor 1 Concentrations in Healthy Cats and Cats with Diabetes Mellitus or Other Diseases. Am. J. Vet. Res..

[22] Harr K.E., Flatland B., Nabity M., Freeman K.P. (2013). ASVCP Guidelines: Allowable Total Error Guidelines for Biochemistry. Vet. Clin. Pathol..

[23] Strage E.M., Theodorsson E., Ström Holst B., Lilliehöök I., Lewitt M.S. (2015). Insulin-like Growth Factor I in Cats: Validation of an Enzyme-Linked Immunosorbent Assay and Determination of Biologic Variation. Vet. Clin. Pathol..

[24] Bidlingmaier M., Friedrich N., Emeny R.T., Spranger J., Wolthers O.D., Roswall J., Körner A., Obermayer-Pietsch B., Hübener C., Dahlgren J. (2014). Reference Intervals for Insulin-like Growth Factor-1 (IGF-I) from Birth to Senescence: Results from a Multicenter Study Using a New Automated Chemiluminescence IGF-I Immunoassay Conforming to Recent International Recommendations. J. Clin. Endocrinol. Metab..

[25] Siemens GmbH (2018). IMMULITE 2000 IGF-1 (Manufacturer Instruction).

[26] Ricós C., Alvarez V., Cava F., García-Lario J.V., Hernández A., Jiménez C.V., Minchinela J., Perich C., Simón M. (1999). Current Databases on Biological Variation: Pros, Cons and Progress. Scand. J. Clin. Lab. Investig..

[27] European Medicines Agency (EMA) (2011). Guideline on Bioanalytical Method Validation.

[28] Flatland B., Freeman K.P., Friedrichs K.R., Vap L.M., Getzy K.M., Evans E.W., Harr K.E. (2010). Quality Assurance Guidelines: Control of General Analytical Factors in Veterinary Laboratories. Vet. Clin. Pathol..

[29] Reusch C.E., Kley S., Casella M., Nelson R.W., Mol J., Zapf J. (2006). Measurements of Growth Hormone and Insulin-like Growth Factor 1 in Cats with Diabetes Mellitus. Vet. Rec..

[30] Westgard J. https://www.westgard.com/lesson42.html.

[31] Passing H., Bablok W. (1983). A New Biometrical Procedure for Testing the Equality of Measurements from Two Different Analytical Methods. Application of Linear Regression Procedures for Method Comparison Studies in Clinical Chemistry, Part I. J. Clin. Chem. Clin. Biochem..

[32] Passing H., Bablok W. (1984). Comparison of Several Regression Procedures for Method Comparison Studies and Determination of Sample Sizes. Application of Linear Regression Procedures for Method Comparison Studies in Clinical Chemistry, Part II. J. Clin. Chem. Clin. Biochem..

[33] Geffré A., Concordet D., Braun J.-P., Trumel C. (2011). Reference Value Advisor: A New Freeware Set of Macroinstructions to Calculate Reference Intervals with Microsoft Excel. Vet. Clin. Pathol..

[34] Raiman Y., Kooistra H., Mol I.J., Prins M. (2014). Reference Range of the IGF-1 Concentration in Feline Plasma Using an Immunochemiluminescence Assay. Master’s Thesis.

[35] Soldin O.P., Dahlin J.R.B., Gresham E.G., King J., Soldin S.J. (2008). IMMULITE^®^ 2000 Age and Sex-Specific Reference Intervals for Alpha Fetoprotein, Homocysteine, Insulin, Insulin-like Growth Factor-1, Insulin-like Growth Factor Binding Protein-3, C-Peptide, Immunoglobulin E and Intact Parathyroid Hormone. Clin. Biochem..

[36] Quimby J., Gowland S., Carney H.C., DePorter T., Plummer P., Westropp J. (2021). 2021 AAHA/AAFP Feline Life Stage Guidelines. J. Feline Med. Surg..

[37] Zini E., Salesov E., Willing A., Palizzotto C., Lutz T.A., Reusch C.E. (2021). Serum Insulin-like Growth Factor-1 Concentrations in Healthy Cats before and after Weight Gain and Weight Loss. J. Vet. Intern. Med..

[38] Kienzle E., Moik K. (2011). A Pilot Study of the Body Weight of Pure-Bred Client-Owned Adult Cats. Br. J. Nutr..

[39] König M.L., Henke D., Adamik K., Vera C.P. (2018). Juvenile Hyposomatotropism in a Somali Cat Presenting with Seizures Due to Intermittent Hypoglycaemia. J. Feline Med. Surg. Open Rep..

[40] Donaldson D., Billson F.M., Scase T.J., Sparkes A.H., McConnell F., Mould J.R.B., Adams V. (2008). Congenital Hyposomatotropism in a Domestic Shorthair Cat Presenting with Congenital Corneal Oedema. J. Small Anim. Pract..

[41] Strage E.M., Sundberg M., Holst B.S., Andersson Franko M., Ramström M., Fall T., Lewitt M. (2018). Effect of Insulin Treatment on Circulating Insulin-like Growth Factor I and IGF-Binding Proteins in Cats with Diabetes Mellitus. J. Vet. Intern. Med..

[42] Steele M.M.E., Borgeat K., Payne J.R., Coss P., Navarro-Cubas X., Church D.B., Niessen S.J.M., Connolly D.J. (2021). Increased Insulin-like Growth Factor 1 Concentrations in a Retrospective Population of Non-Diabetic Cats Diagnosed with Hypertrophic Cardiomyopathy. J. Feline Med. Surg..

[43] Fletcher J.M., Scudder C.J., Kiupel M., Pipe-Martin H.N., Kenny P.J., Mantis P., Fenn J., Smith K., Blair R.V., Granger L.A. (2016). Hypersomatotropism in 3 Cats without Concurrent Diabetes Mellitus. J. Vet. Intern. Med..

[44] Fracassi F., Salsi M., Sammartano F., Bo S., Kooistra H.S. (2016). Acromegaly in a Non-Diabetic Cat. J. Feline Med. Surg. Open Rep..

