# Supplementary material for: Method Validation and Establishment of Reference Intervals for an Insulin-like Growth Factor-1 Chemiluminescent Immunoassay in Cats
Source: Vet Sci. 2023 Sep 15;10(9):575. doi: 10.3390/vetsci10090575 (PMC10534906; doi:10.3390/vetsci10090575)
Supplement: Supplementary file 1 [file vetsci-10-00575-s001.zip › Table S1.pdf]

Table S1. Results of first and repeated 10 IGF-1 samples measured by radioimmunoassay stored >9 years at -80°C.

Month/year of first measurement	Value (ng/ml)	Month/year of second measurement	Value (ng/ml)	Mean (ng/ml)	SD	CV (%)
11/2011	1813	02/2021	1533	1673	140	8.37
06/2011	1736	02/2021	1779	1757.5	21.5	1.22
06/2011	1321	02/2021	1356	1338.5	17.5	1.31
12/2011	1302	02/2021	1591	1446.5	144.5	9.99
01/2010	1907	02/2021	2001	1954	47	2.41
10/2011	1531	02/2021	1579	1555	24	1.54
01/2012	1442	02/2021	1761	1601.5	159.5	9.96
01/2010	842	02/2021	857	849	7.5	0.88
06/2012	947	02/2021	852	899.5	47.5	5.28
02/2012	896	02/2021	956	930.5	34.5	3.71

Disclaimer/Publisher's Note: The statements, opinions and data contained in all publications are solely those of the individual author(s) and contributor(s) and not of MDPI and/or the editor(s). MDPI and/or the editor(s) disclaim responsibility for any injury to people or property resulting from any ideas, methods, instructions or products referred to in the content.
